# Mutations in the UQCC1-Interacting Protein, UQCC2, Cause Human Complex III Deficiency Associated with Perturbed Cytochrome *b* Protein Expression

**DOI:** 10.1371/journal.pgen.1004034

**Published:** 2013-12-26

**Authors:** Elena J. Tucker, Bas F. J. Wanschers, Radek Szklarczyk, Hayley S. Mountford, Xiaonan W. Wijeyeratne, Mariël A. M. van den Brand, Anne M. Leenders, Richard J. Rodenburg, Boris Reljić, Alison G. Compton, Ann E. Frazier, Damien L. Bruno, John Christodoulou, Hitoshi Endo, Michael T. Ryan, Leo G. Nijtmans, Martijn A. Huynen, David R. Thorburn

**Affiliations:** 1 Murdoch Children's Research Institute, Royal Children's Hospital, Parkville, Victoria, Australia; 2 Department of Paediatrics, University of Melbourne, Melbourne, Victoria, Australia; 3 Centre for Molecular and Biomolecular Informatics, Nijmegen Centre for Molecular Life Sciences, Radboud University Medical Centre, Nijmegen, The Netherlands; 4 Nijmegen Center for Mitochondrial Disorders, Radboud University Medical Centre, Nijmegen, The Netherlands; 5 Department of Biochemistry, La Trobe Institute for Molecular Science, La Trobe University, Melbourne, Victoria, Australia; 6 Victorian Clinical Genetics Services, Royal Children's Hospital, Melbourne, Victoria, Australia; 7 Genetic Metabolic Disorders Research Unit, Children's Hospital at Westmead, Westmead, New South Wales, Australia; 8 Disciplines of Paediatrics & Child Health and Genetic Medicine, University of Sydney, Sydney, New South Wales, Australia; 9 Department of Biochemistry, Jichi Medical University, Tochigi, Japan; 10 ARC Centre of Excellence for Coherent X-ray Science, La Trobe University, Melbourne, Australia; University of Miami, United States of America

## Abstract

Mitochondrial oxidative phosphorylation (OXPHOS) is responsible for generating the majority of cellular ATP. Complex III (ubiquinol-cytochrome *c* oxidoreductase) is the third of five OXPHOS complexes. Complex III assembly relies on the coordinated expression of the mitochondrial and nuclear genomes, with 10 subunits encoded by nuclear DNA and one by mitochondrial DNA (mtDNA). Complex III deficiency is a debilitating and often fatal disorder that can arise from mutations in complex III subunit genes or one of three known complex III assembly factors. The molecular cause for complex III deficiency in about half of cases, however, is unknown and there are likely many complex III assembly factors yet to be identified. Here, we used Massively Parallel Sequencing to identify a homozygous splicing mutation in the gene encoding Ubiquinol-Cytochrome *c* Reductase Complex Assembly Factor 2 (UQCC2) in a consanguineous Lebanese patient displaying complex III deficiency, severe intrauterine growth retardation, neonatal lactic acidosis and renal tubular dysfunction. We prove causality of the mutation via lentiviral correction studies in patient fibroblasts. Sequence-profile based orthology prediction shows UQCC2 is an ortholog of the *Saccharomyces cerevisiae* complex III assembly factor, Cbp6p, although its sequence has diverged substantially. Co-purification studies show that UQCC2 interacts with UQCC1, the predicted ortholog of the Cbp6p binding partner, Cbp3p. Fibroblasts from the patient with *UQCC2* mutations have deficiency of UQCC1, while UQCC1-depleted cells have reduced levels of UQCC2 and complex III. We show that UQCC1 binds the newly synthesized mtDNA-encoded cytochrome *b* subunit of complex III and that UQCC2 patient fibroblasts have specific defects in the synthesis or stability of cytochrome *b*. This work reveals a new cause for complex III deficiency that can assist future patient diagnosis, and provides insight into human complex III assembly by establishing that UQCC1 and UQCC2 are complex III assembly factors participating in cytochrome *b* biogenesis.

## Author Summary

Mitochondrial complex III deficiency is a devastating disorder that impairs energy generation, and leads to variable symptoms such as developmental regression, seizures, kidney dysfunction and frequently death. The genetic basis of complex III deficiency is not fully understood, with around half of cases having no known cause. This lack of genetic diagnosis is partly due to an incomplete understanding of the genes required for complex III assembly and function. We have identified two key proteins required for complex III, UQCC1 and UQCC2, and have elucidated the role of these inter-dependent proteins in the biogenesis of cytochrome *b*, the only complex III subunit that is encoded by mitochondrial DNA. We have shown that mutations in *UQCC2* cause human complex III deficiency in a patient with neonatal lactic acidosis and renal tubulopathy. This work contributes to an improved understanding of complex III biogenesis, and will aid future molecular diagnoses of complex III deficiency.

## Introduction

Mitochondrial disorders of ubiquinol-cytochrome *c* oxidoreductase (complex III, MIM 124000) represent a significant proportion of patients with OXPHOS dysfunction [1], [2]. Their identification is challenging due to (a) the sheer number of candidate genes, (b) their complicated interplay and (c) an incomplete understanding of complex III assembly. To date, mutations in only eight human genes have been identified as responsible for complex III deficiency. The first mutation was identified in the only mtDNA-encoded subunit of complex III, cytochrome *b* (*MT-CYB*, MIM 516020), in an adult patient with progressive exercise intolerance [3]. Patients with *MT-CYB* mutations have since been reported with a range of phenotypes and symptoms including Mitochondrial Encephalomyopathy, Lactic Acidosis and Stroke-like episodes (MELAS, MIM 540000), mitochondrial myopathy, cardiomyopathy and multisystem failure [2], [4]. While mtDNA mutations are the most common identified cause of complex III deficiency, mutations in nuclear genes can also be causative. Mutations have been reported in four nuclear genes encoding complex III subunits; the supernumerary subunits UQCRB (MIM 191330) [5] and UQCRQ (MIM 612080) [6] and more recently the core subunit UQCRC2 (MIM 191329) [7] and the catalytic subunit CYC1 (MIM 123980) [8]. Patients with *UQCRB* and *UQCRC2* mutations presented with hypoglycaemia and lactic acidosis, the patient with *UQCRQ* mutations presented with severe psychomotor retardation and patients with *CYC1* mutations presented with recurrent ketoacidosis and insulin-responsive hyperglycemia [8]. In addition to complex III subunit genes, mutations have also been identified in two complex III assembly factor genes, *BCS1L* (MIM 603647) [9], *TTC19* (MIM 613814) [10] and recently *LYRM7* [11]. *BCS1L* mutations were first identified in 2001 [9] and since then more than 20 additional mutations of the *BCS1L* gene have been reported [2]. The clinical presentation of patients with *BCS1L* mutations varies greatly with some mutations being associated with tubulopathy, encephalopathy and liver failure [9], others with GRACILE syndrome (growth retardation, aminoaciduria, cholestasis, iron overload, lactic acidosis and early death, MIM 603358) [12], with isolated encephalopathy [13] or with Björnstad syndrome characterized by sensorineural deafness associated with short brittle hair (MIM 262000) [14]. Mutations in *TTC19* have been shown to cause encephalopathy with variable age of onset and rate of progression, which in some patients is associated with severe psychiatric manifestations [10], [15]. Mutations in *LYRM7* are associated with early onset encephalopathy [11]. Despite the discovery of pathogenic mutations in eight different complex III-related genes, the majority of patients with complex III deficiency remain unsolved.

We aimed to identify new genes underpinning complex III deficiency and to elucidate their role in the complex III assembly process. We identified a causative homozygous *UQCC2* (MIM 614461) splicing mutation in a patient with severe intrauterine growth retardation, neonatal lactic acidosis and renal tubular dysfunction associated with complex III deficiency. We established the role of UQCC2 as a complex III assembly factor that cooperates with UQCC1 (MIM 611797) to mediate cytochrome *b* protein expression and subsequent complex III assembly.

## Results

### MitoExome sequencing identified a likely deleterious homozygous mutation in UQCC2

**Figure 1:**
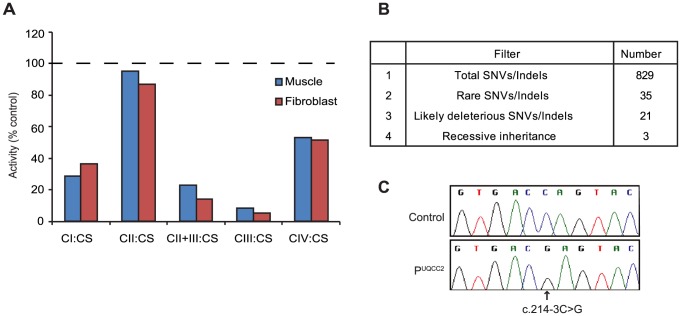
MitoExome sequencing identifies a homozygous mutation in *UQCC2* in a patient with complex III deficiency. (A) The activity of complexes I–IV (CI-IV) as measured by spectrophotometric analysis and normalized to the activity of citrate synthase (CS), expressed as a percentage of control. Values are the average of duplicate assays. (B) Prioritization of single nucleotide variants (SNVs) and small insertion/deletions (indels) identified by MitoExome MPS. (C) Sequence chromatograms of *UQCC2* in control and patient gDNA validating the c.214-3C>G mutation detected by MitoExome sequencing.

We studied a consanguineous Lebanese patient presenting with severe intrauterine growth retardation, neonatal lactic acidosis and renal tubular dysfunction. Spectrophotometric enzyme assays revealed a severe complex III deficiency with residual activity of only 9% in skeletal muscle and 5% in skin fibroblasts when normalized to citrate synthase activity (Figure 1A). Complex I activity was reduced to 29% and 37% and complex IV activity was reduced to 51% and 53% in muscle and fibroblasts respectively, whereas complex II activity was normal. Secondary deficiency in complex I and complex IV have been described in patients with primary complex III deficiency previously [16]–[19]. In our own experience, skeletal muscle from 4 previous patients with pathogenic mutations in genes encoding complex III subunits or assembly factors (*CYC1*, *UQCRC2*, and two *BCS1L*) had residual activities for complexes I, II, III and IV of 54±17, 117±17, 16±6 and 55±13 (expressed as % of control mean relative to citrate synthase; mean ± S.E.M.). Given the severity of the complex III defect in regard to the activities of complexes I and IV, we thus regarded the patient as having a primary complex III defect. In order to uncover the molecular basis for the complex III defect, we performed “MitoExome” sequencing, involving the targeted capture and massively parallel sequencing (MPS) of the mtDNA and ∼1000 nuclear genes predicted to encode the entire mitochondrial proteome [20]. This approach identified 829 single nucleotide variants or small insertion/deletions in the patient, which were analyzed to select the disease gene (Figure 1B). Three genes, *TYMP*, *MTCH1* and *UQCC2*, harbored rare (allele frequency <0.005 in dbSNP version 132 [21] and the 1000 genomes project release 20100804 [22]) homozygous or compound heterozygous variants that were predicted to potentially impact protein function. Although *TYMP* is a known OXPHOS “disease gene”, the clinical and biochemical presentation of the patient, plus the consanguinity, suggested that the rare compound heterozygous *TYMP* mutations (c.242G>A, c.148A>C) were not causal. Patients with *TYMP* mutations typically present with combined OXPHOS deficiency associated with mtDNA deletions/depletion rather than a primary complex III deficiency, and have mitochondrial neurogastrointestinal encephalomyopathy (MNGIE, MIM 603041) as opposed to the primary lactic acidosis and renal tubulopathy observed in our patient [23]. Although the homozygous c.170C>T mutation in the pro-apoptotic *MTCH1* was not reported in dbSNP version 132 [21] or the 1000 genomes project release 20100804 [22], it was detected with a minor allele frequency of 0.005526 in the Exome Variant Server (NHLBI GO Exome Sequencing Project, http://evs.gs.washington.edu/EVS/ March 2013), suggesting it is likely to be too common to cause a rare mitochondrial disorder. Computational analyses suggested that the third candidate, *UQCC2* (previously called *MNF1*, *M19* or *C6orf125*) was likely causal based on 1) its orthology to the *S. cerevisiae* complex III assembly factor Cbp6p, and 2) its co-expression with complex III subunits (see below). Further analyses indicated a wider conservation of complex III assembly factors, as the interaction partner of *S. cerevisiae* Cbp6p, Cbp3p, also had a mammalian homolog, UQCC1 (previously called UQCC), which was co-expressed with *UQCC2* and similarly co-expressed with complex III subunit genes. The homozygous c.214-3C>G *UQCC2* (NM_032340) mutation fell within a 30.7 Mb long contiguous stretch of homozygosity (LCSH) (Figure S1), consistent with both alleles being inherited from a common ancestor. The mutation was verified via Sanger sequencing (Figure 1C) and was not reported in dbSNP version 132 [21], the 1000 genomes project release 20100804 [22] or the Exome Variant Server. To investigate whether the c.214-3C>G mutation might be a common variant found within the Lebanese population, a Sequenom assay was developed to genotype 86 Lebanese controls. The c.214-3C>G variant was not detected, suggesting it is rare in the ethnically-matched population. To see if this gene might be the cause of complex III deficiency in other patients, we sequenced the coding regions of *UQCC2* in 11 patients with confirmed complex III deficiency who lacked a molecular diagnosis. No potentially pathogenic changes were identified.

### The c.214-3C>G mutation causes a severe defect in UQCC2 splicing

**Figure 2:**
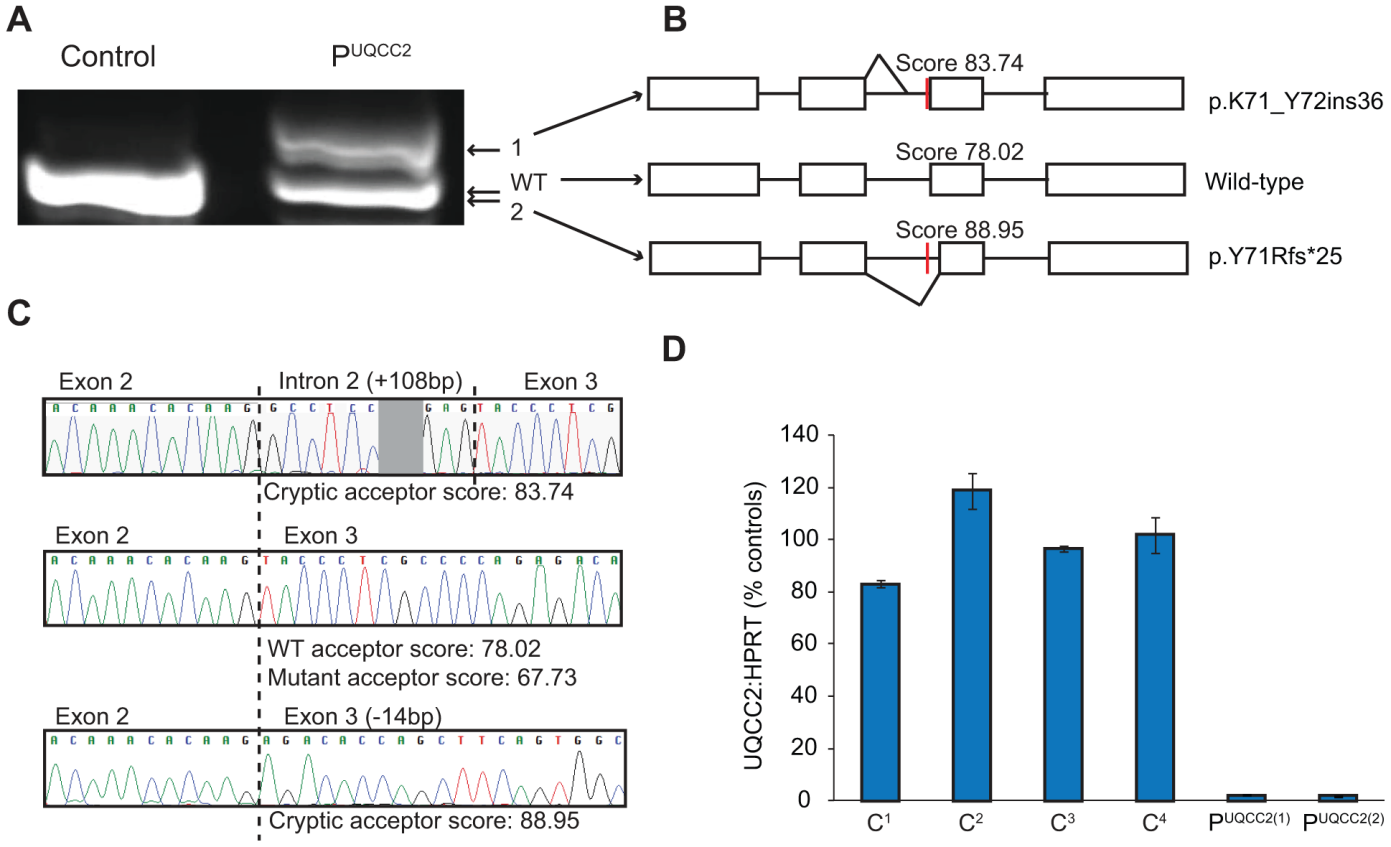
The c.214-3C>G mutation causes a severe *UQCC2* splicing defect. (A) Gel electrophoresis of full-length *UQCC2* RT-PCR products from fibroblasts grown in the absence of cycloheximide. Two prominent bands are seen in P^UQCC2^ whereas only one is observed in the control. (B) Schematic diagram shows the wild-type (WT) mRNA structure and the two splice variants (1 and 2) observed in P^UQCC2^. (C) Sequence chromatograms of cloned RT-PCR products show that the upper product in P^UQCC2^ retains 108 bases of intronic sequence due to the use of a cryptic acceptor site, and that the lower product in P^UQCC2^ lacks 14 bases of exonic sequence due to the use of an alternative cryptic acceptor site. Splice site prediction scores are from Human Splicing Finder v2.4.1 (http://www.umd.be/HSF/). (D) qRT-PCR analysis using an assay that detects the exon 2/3 junction of *UQCC2* (normalized to the endogenous control *HPRT1*) demonstrates P^UQCC2^ fibroblasts have only 2% wild-type *UQCC2* expression relative to controls (C^1^–C^4^). P^UQCC2(1)^ and P^UQCC2(2)^ represent separate fibroblast subcultures.

The c.214-3C>G *UQCC2* mutation is found 3 bases upstream of exon 3. The third base upstream of exons generally has only moderate conservation, usually being a cytosine or a thymine and never a guanine [24]. In keeping with this, the c.214-3C>G *UQCC2* site is moderately conserved, with no vertebrate having a guanine at this position (Figure S2). To investigate whether the c.214-3C>G mutation causes a splicing defect, Reverse Transcriptase (RT)-PCR was performed using RNA extracted from patient fibroblasts. Sequencing revealed aberrant mRNA splicing in the patient, with two major mRNA splice variants (Figure 2A), both of which were generated by the use of cryptic acceptor sites (Figure 2B–C and Text S1). Patient fibroblasts had only 2% residual wild-type *UQCC2* expression (Figure 2D), as determined by qRT-PCR, suggesting the c.214-3C>G mutation almost completely abolishes wild-type splicing. In keeping with this, there was no detectable UQCC2 protein observed by western blot (Figure S3). The protein encoded by the alternative splice species is likely unstable, as there was also no evidence of elongated or truncated UQCC2 protein (Figure S3).

### UQCC1 and UQCC2 are putative complex III assembly factors

**Figure 3:**
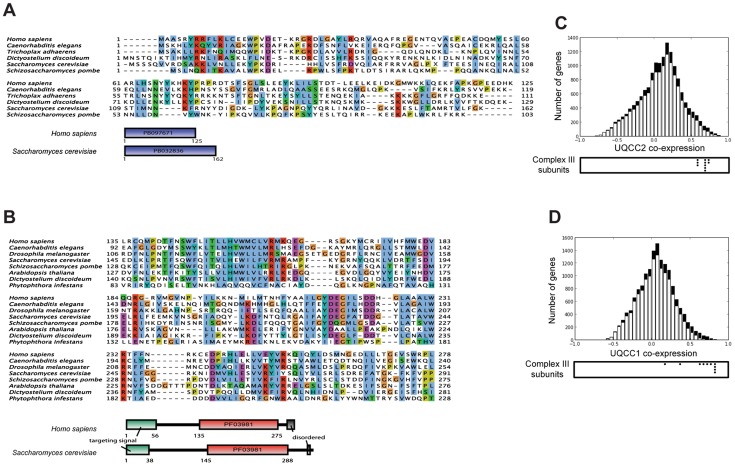
UQCC2 and UQCC1 are orthologous to the fungal complex III assembly factors Cbp6p and Cbp3p. Alignment between fungal and human complex III assembly factors was inferred using iterative orthology pipeline Ortho-Profile [26] and visualized using JalView with the ClustalX color scheme [65]. (A) Alignment of conserved regions among the orthologs of human UQCC2 and fungal Cbp6p. The *S. cerevisiae*-specific insertion between residues 47 and 96 is replaced with a letter X. The sequences do not have a recognizable targeting signal or additional conserved motifs. Domains were annotated according to PFAM [66]. (B) Alignment of UQCC1 (human) and Cbp3p (yeast) with orthologs in other eukaryotes. Only the conserved part of the sequence is shown in the alignment. Proteins contain the UQCC1-specific domain PF03981. (C and D) Mouse mRNA co-expression of *UQCC2* (C) and *UQCC1* (D) with other genes across 91 murine cell types and tissues. Black bars represent genes encoding mitochondrial proteins and white bars represent the remaining human genes. Below the chart the co-expression values of complex III subunits are indicated with black dots.

Human UQCC2 was previously postulated to have a role in mtDNA maintenance and was found to associate with mitochondrial nucleoids [25], however, PicoGreen staining indicated mitochondrial nucleoids were not disturbed in patient fibroblasts (Figure S4A). The patient also had no significant mtDNA depletion, having 78% mtDNA compared to the mean of 4 control fibroblast cell lines when estimated by qPCR analysis (Figure S4B). Iterative orthology prediction using the Ortho-Profile method [26] revealed that the *UQCC2* gene is an ortholog of the *S. cerevisiae CBP6* gene that is required for complex III assembly [27] (Figure 3A). We used the Ortho-Profile method to investigate whether there was wider conservation of complex III assembly factors and found that Cbp3p, which cooperates with Cbp6p in complex III assembly, also has a predicted human ortholog, UQCC1 (Figure 3B). Both orthologous groups have diverged significantly among eukaryotes with UQCC2/Cbp6p having an overall low amino acid conservation (Figure 3A), while only the C-terminus of UQCC1/Cbp3p is conserved (human residues 135–279) (Figure 3B). The N-terminal regions of UQCC1/Cbp3p proteins (residues 1–134 in human and 1–144 in yeast) are highly divergent in both metazoa and fungi, with homologous sequences recognizable only in closely related species (vertebrates for the N-terminal fragment of UQCC1 and the Saccharomyceta clade for Cbp3p). Interestingly, the amino acids from positions 12 to 96 have been shown to be relatively dispensable for Cbp3p function, explaining the lack of sequence conservation [28]. To support the association of UQCC1 and UQCC2 proteins with complex III in mammals, we investigated the co-expression of the genes with complex III subunit genes in 91 mouse tissues and cell types. *UQCC1* and *UQCC2* genes co-express highly with each other at the mRNA level (Pearson correlation 0.636). Genes for complex III subunits co-express significantly with both *UQCC2* (average 0.66, Figure 3C) and *UQCC1* (average 0.66, Figure 3D). The co-expression of the two genes with complex III subunits is, on average, 3-fold higher than with genes encoding other mitochondrial proteins (two-sided Mann–Whitney test P-value <2×10^−5^). Both genes are conserved in amoebozoa indicating their ancient evolutionary origin, preceding the divergence of human and fungi (Figure 3A and 3B). UQCC1 is additionally conserved in distantly related eukaryotes that contain mitochondria, including the stramenopile *Phytophthora infestans*. A related stramenopile, *Blastocystis hominis*, has lost respiratory chain complexes III, IV and V and congruently we did not identify orthologs of the two complex III assembly factors in its genome.

### The UQCC2 mutation is responsible for complex III deficiency

**Figure 4:**
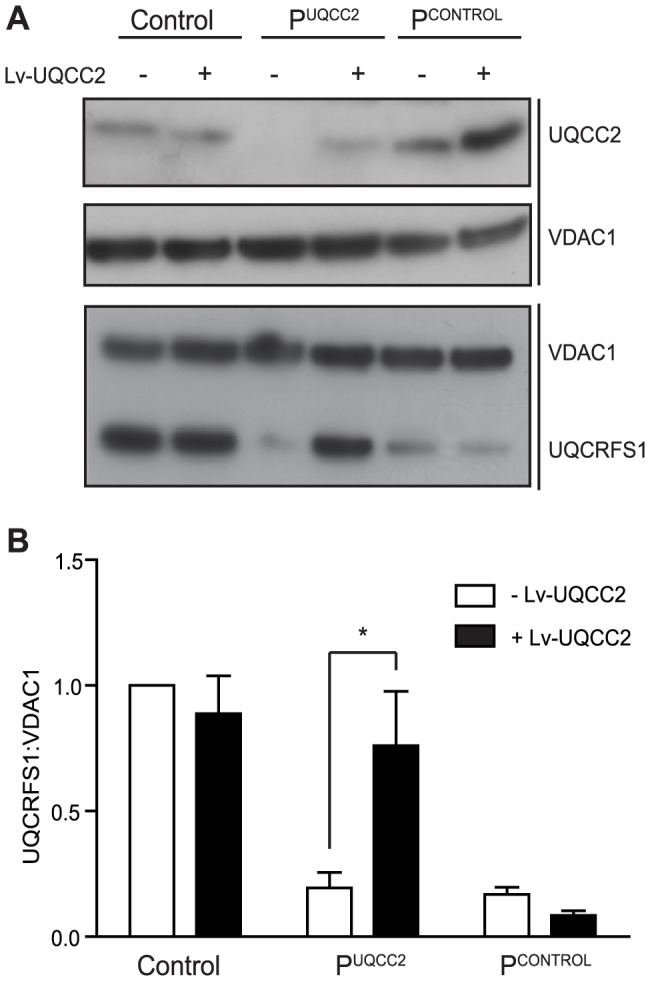
*UQCC2* mutations are responsible for the complex III defect in P^UQCC2^. Fibroblasts from Control, P^UQCC2^ with mutations in *UQCC2* and P^CONTROL^ with mutations in a complex III subunit gene (and no *UQCC2* mutation) were transduced with wild-type *UQCC2* mRNA. (A) Representative SDS-PAGE western blot shows reduced UQCC2 in P^UQCC2^ and increased UQCC2 expression following *UQCC2* transduction. VDAC1 acts as a loading control. UQCRFS1 protein is reduced in both complex III deficient patients and restored in P^UQCC2^, but not P^CONTROL^, with *UQCC2* transduction. VDAC1 acts as a loading control. (B) The intensity of immunostained UQCRFS1 relative to VDAC1 before and after transduction with *UQCC2* was quantified by densitometry. Error bars indicate 1 s.e.m. for 3 independent transductions and the asterisk indicates p<0.05, two way ANOVA.

To verify that the *UQCC2* mutation was indeed responsible for the complex III defect, patient fibroblasts were transduced with a lentiviral construct expressing wild-type *UQCC2* mRNA to examine whether it could restore complex III assembly. Lentiviral transduction caused UQCC2 expression in P^UQCC2^ to increase to a level comparable to controls (Figure 4A). To assess complex III restoration, western blotting for the complex III subunit UQCRFS1 was performed as this subunit was clearly degraded in patient fibroblasts (Figure 4). As a negative control, fibroblasts with a mutation in a complex III subunit (manuscript in preparation) but wild-type *UQCC2* were transduced in parallel. Lentiviral transduction of *UQCC2* caused a significant increase in UQCRFS1 protein expression in the patient with *UQCC2* mutations (p<0.05, Two-Way ANOVA), but caused no significant change in the normal control or the complex III deficient patient with mutations in a complex III subunit gene. After transduction, the level of UQCRFS1 protein expression in P^UQCC2^ was no longer significantly different from control cells. We also measured complex III activity in fibroblasts before and after transduction with *UQCC2*. There was a clear increase in complex III activity after transduction with *UQCC2* in P^UQCC2^ from 29% to 73% of control (Figure S5A). These results prove that the complex III deficiency is due to a lack of UQCC2, and that the mutations in other candidate genes in the patient, *TYMP* and *MTCH1*, are not causative.

### UQCC2 deficiency is associated with disturbed complex III assembly

**Figure 5:**
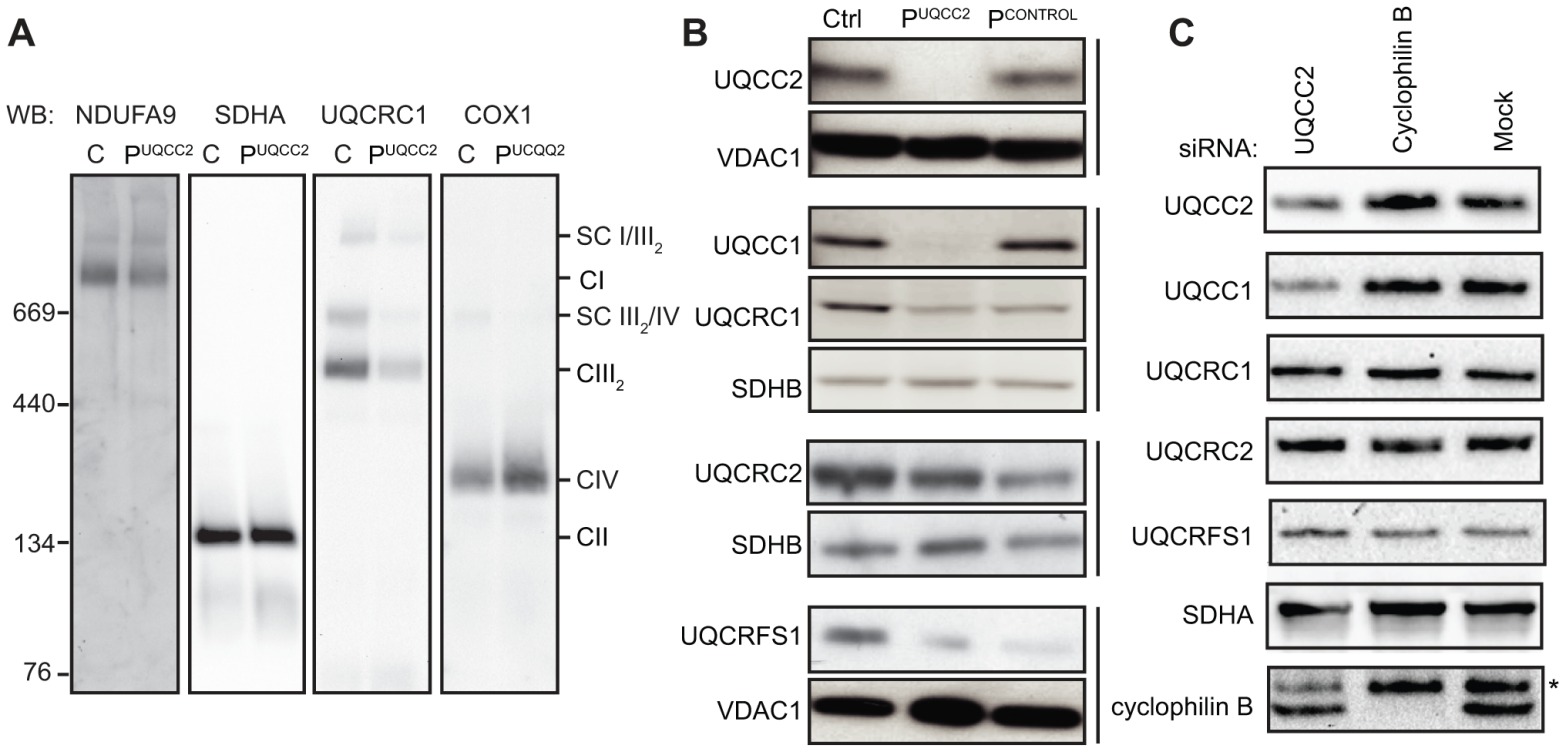
Lack of UQCC2 is associated with aberrant complex III assembly, subunit expression and UQCC1 stability. (A) BN-PAGE immunoblotting of mitochondria lysed in 1% Triton X-100, using antibodies against the NDUFA9 subunit of complex I, the SDHA subunit of complex II, the UQCRC1 subunit of complex III and the COX1 subunit of complex IV shows reduced complex III assembly in P^UQCC2^. See Figure S6A for quantification of immunoreactive bands. (B) SDS-PAGE and western blotting of mitochondrial lysates from patient fibroblasts demonstrate a marked deficiency of UQCC2 and UQCC1, a mild deficiency in the UQCRC2 subunit of complex III, and a more pronounced deficiency of the UQCRC1 and UQCRFS1 subunits of complex III. The P^CONTROL^ cell line with mutations in a complex III subunit gene showed a similar profile of complex III subunit instability but had levels of UQCC2 and UQCC1 comparable to the wild-type control. The complex II subunit SDHB and mitochondrial VDAC1 protein act as loading controls. Vertical bars indicate immunoblots performed using the same membrane. See Figure S6A for quantification of immunoreactive bands. (C) Mitochondrial lysates of HEK293 cells transfected with siRNA targeting *UQCC2* analyzed by SDS-PAGE and western blotting showed reduced levels of UQCC2 and UQCC1 proteins. As control, *cyclophilin B* knockdown and mock transfected cells were used. The asterisk indicates a non-specific, cross-reactive species. See Figure S7A for quantification of immunoreactive bands.

To further characterize the biochemical consequence of UQCC2 deficiency, protein levels and complex assembly were analyzed by sodium dodecyl sulphate (SDS)-polyacrylamide gel electrophoresis (PAGE) and Blue Native (BN)-PAGE using patient fibroblasts. BN-PAGE and immunoblot analysis of mitochondria lysed with Triton X-100 revealed that P^UQCC2^ had a severe complex III defect, with a markedly reduced amount of complex III holocomplex but normal levels of complex II (Figure 5A and Figure S6A). Consistent with enzyme analysis, there was a moderate reduction in complex I holocomplex, likely due to instability of complex I due to complex III deficiency [17]. Interestingly, despite mildly reduced complex IV activity in patient fibroblasts (Figure 1A), the level of complex IV holoenzyme appeared increased (Figure 5A and Figure S6A). We also analyzed patient complexes by BN-PAGE of mitochondria lysed with digitonin, which allows visualization of OXPHOS supercomplexes. Prior to transduction, the small amount of complex III detectable in P^UQCC2^ appears to be in the supercomplex form with little or no complex III dimer (Figure S5B). However, transduction with *UQCC2* restores the relative amounts of complex III in P^UQCC2^ in the dimer and supercomplex forms to ratios similar to control (Figure S5B), further confirming *UQCC2* as the causative gene. SDS-PAGE analysis demonstrated that fibroblasts from P^UQCC2^ had a mild defect in the level of the complex III subunit, UQCRC2, and a more pronounced deficiency of the UQCRFS1 and UQCRC1 subunits (Figure 5B and Figure S6B). The UQCC2 binding partner, UQCC1, was barely detectable by western blot, suggesting UQCC2 is required for its stability. We confirmed that the lack of UQCC1 is due to the UQCC2 deficiency, by repeating SDS-PAGE analysis on fibroblasts transduced with *UQCC2* (Figure S5C). We also transfected HEK293 cells with an siRNA targeting *UQCC2* that resulted in a 40% knockdown of the UQCC2 protein and 60% reduction in UQCC1 protein, supporting the requirement of UQCC2 for UQCC1 stability (Figure 5C and Figure S7A). In contrast to patient fibroblasts, no obvious defect in complex III subunit levels was observed in cells with *UQCC2* knockdown. This is likely a consequence of the less severe UQCC2 deficiency achieved in knockdown experiments, with 60% residual protein compared to no detectable protein in patient fibroblasts.

### UQCC1 and UQCC2 interact

**Figure 6:**
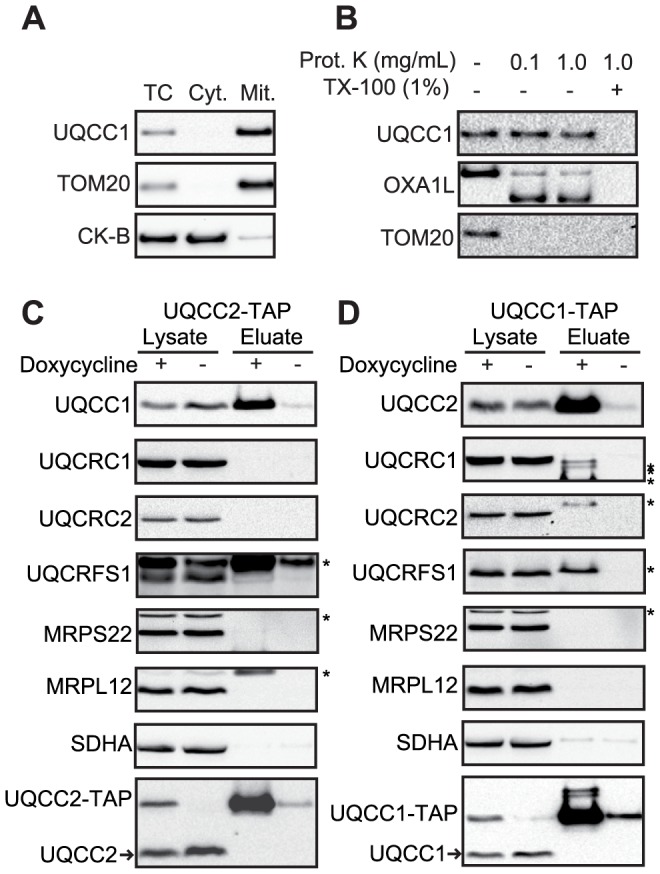
UQCC2 interacts with mitochondrial protein UQCC1. (A) SDS-PAGE analysis of HEK293 cellular fractions shows that UQCC1 is enriched in the mitochondrial fraction, similar to the mitochondrial protein TOM20. A cytosolic marker creatine kinase B-type (CK-B) was used. TC: Total Cell, Cyt: Cytoplasmic fraction, Mit: Mitochondrial fraction. (B) Proteinase K protection assay performed using mitochondria with digitonin-permeabilized outer membranes shows localization of UQCC1 within the mitochondrial inner membrane. UQCC1, unlike outer membrane localized TOM20 and the inter-membrane localized part of OXA1L, is protected from proteolysis and degraded only after the inner membrane is dissolved with Triton X-100. Western blot analysis of single step affinity purified (C) UQCC2- and (D) UQCC1-TAP from doxycycline-induced HEK293 cells shows that UQCC1 co-purifies with UQCC2-TAP and UQCC2 co-purifies with UQCC1-TAP. Additional probing of the membranes for the complex III structural subunits UQCRC1, UQCRC2, UQCRFS1 and mitochondrial ribosomal subunits MRPS22 and MRPL12 did not reveal co-elution of these proteins. Asterisks with these subunits, including the one with UQCRFS1, correspond to bands at different heights that result from previous incubations. Complex II subunit SDHA was used to rule out non-specific protein binding. Non-induced cells were used as control. Antibodies used are indicated at the left. Arrowheads indicate endogenous UQCC1 and UQCC2.

Given that UQCC2 deficiency was associated with loss of UQCC1, we further investigated the relationship between these proteins. We first confirmed that human UQCC1 is a mitochondrial protein by cellular fractionation and SDS-PAGE (Figure 6A). Proteinase K digestion indicated that UQCC1 localizes to the inner mitochondrial membrane (Figure 6B). In *S. cerevisiae*, the UQCC1 ortholog Cbp3p interacts with the UQCC2 ortholog, Cbp6p, and together they activate translation of mtDNA-encoded cytochrome *b*, bind and stabilize the newly synthesized protein and deliver it to an early complex III assembly intermediate [29], [30]. To address a possible association between UQCC1 and UQCC2, HEK293 cells expressing C-terminal TAP-tagged UQCC1 or UQCC2 under the control of a doxycycline-inducible promoter were generated and subjected to single step affinity purifications. Subsequent SDS-PAGE and western blot analysis of the UQCC2-TAP purification revealed efficient co-isolation of UQCC1 (Figure 6C). The complex III subunits UQCRC1, UQCRC2, UQCRFS1 and the mitochondrial ribosomal subunits MRPL12 and MRPS22 did not co-elute with UQCC2-TAP (Figure 6C). Using a UQCC1-TAP tagged construct we confirmed the interaction with UQCC2 (Figure 6D).

### UQCC1 deficiency is associated with disturbed complex III assembly

**Figure 7:**
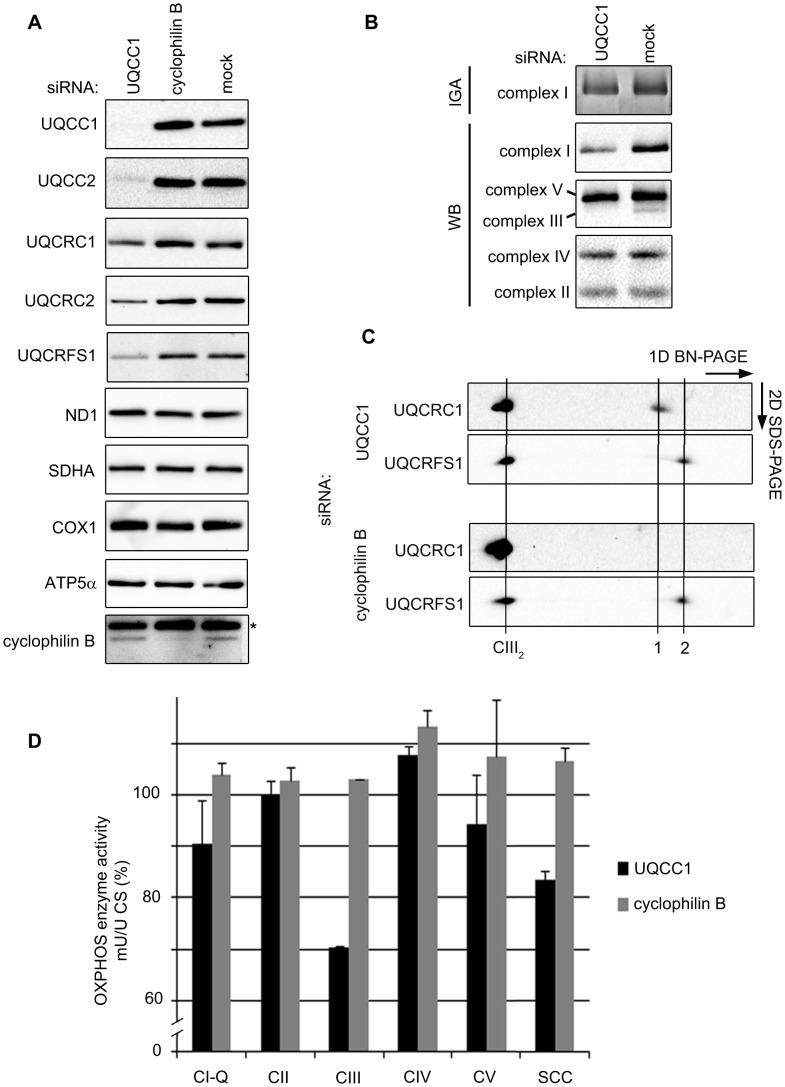
Depletion of the UQCC2 binding partner, UQCC1, affects complex III assembly. (A) SDS-PAGE and western blot analysis of mitochondrial extracts from HEK293 cells transfected with *UQCC1* siRNA shows lower levels of complex III subunits UQCRFS1, UQCRC1 and UQCRC2. Subunits of complex I (ND1), complex II (SDHA), complex IV (COX1) and complex V (ATP5α) are not affected by *UQCC1* knockdown. (B) BN-PAGE of HEK293 cells transfected with UQCC1 siRNA show reduced levels of holocomplex III (UQCRC2) and a mild effect on complex I in gel activity (IGA) and complex I holocomplex levels (NDUFA9). Levels of other OXPHOS complexes, complex II (SDHB), complex IV (COX2) and complex V (ATP5α) are not affected. Mock transfected cells were used as control). See Figure S7B-C for the quantification of the immunoreactive bands. (C) 2D BN-PAGE of HEK293 cells depleted of UQCC1 or cyclophilin B with indicated antibodies. The holocomplex III dimer is indicated with a line labeled CIII_2_. To the right are lower molecular weight subcomplexes: UQCRC1-containing subcomplex (1) and, likely, monomeric UQCRFS1 (2). Lauryl maltoside was used to solubilize OXPHOS complexes in parts B and C. (D) Respiratory chain enzyme activity measurements of HEK293 cells transfected with *UQCC1* and *cyclophilin B* siRNAs. Mock transfected cells were set at 100%. Error bars indicate one standard deviation. Complex I ubiquinone reducing part (CI-Q), complexes II–V (CII-V) and combined activity of complex II and III (SCC) were measured relative to the activity of citrate synthase (CS).

To investigate whether UQCC1-deficient cells exhibit a similar biochemical phenotype to UQCC2-deficient cells, we transfected HEK293 cells with siRNA targeting *UQCC1* or *cyclophilin B*, which, along with mock-transfected cells, served as a negative control. The knockdown led to disappearance of UQCC1 protein in mitochondrial lysates and a concurrent loss of UQCC2 (Figure 7A). Although UQCC2 stability appears to depend on UQCC1, we did not observe that over-expression of either UQCC2-TAP or UQCC1-TAP led to an increase of UQCC1 or UQCC2 respectively (Figure 6C and Figure 6D). Knockdown of UQCC1 led to reduced levels of the UQCRFS1, UQCRC1 and UQCRC2 subunits of complex III (Figure 7A and Figure S7B). With respect to the presence of individual subunits, the impact of *UQCC1* knockdown appears to be limited to complex III, as subunits from complexes I, II, IV and V were unaffected (Figure 7A, Figure 7B and Figure S8). Nevertheless, with respect to the presence of complete OXPHOS complexes, as measured with BN-PAGE analysis, we do observe besides a marked reduction of complex III, also a reduction of complex I (Figure 7B and Figure S7C). The *UQCC1* knockdown profile is thus similar to UQCC2 deficiency in P^UQCC2^, with reduced levels of complex III subunits, a reduced level of complex III and, to a lesser extent, a reduced level of complex I (Figure 5A, 5B).

Two-dimensional BN-PAGE experiments confirmed lower levels of mature complex III and additionally showed the accumulation of a partially assembled subcomplex containing UQCRC1. This subcomplex was not detected in the *cyclophilin B* knockdown control cells (Figure 7C). Subsequent OXPHOS enzyme activity measurements of UQCC1-depleted cells showed reduced complex III, a reduced combined complex II/III activity (SCC) and a slight but considerable reduction in complex I, compared to the mock control (Figure 7D).

### UQCC1 and UQCC2 are required for cytochrome b protein expression

**Figure 8:**
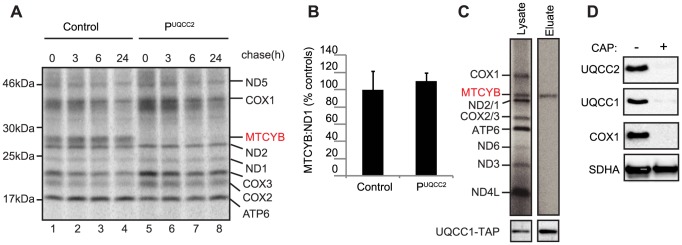
UQCC2 and UQCC1 are involved in cytochrome *b* translation and/or stability. (A) SDS-PAGE analysis of ^35^S-methionine-labeled mtDNA-encoded proteins in patient fibroblasts shows a lack of cytochrome *b* (MTCYB) protein (even at zero hours chase) suggesting a defect in cytochrome *b* synthesis or its immediate stability. (B) qRT-PCR shows normal expression of *cytochrome b* (*MTCYB*) mRNA in patient fibroblasts. (C) Autoradiogram of single step affinity purified UQCC1-TAP with ^35^S metabolically labeled mitochondrial translation products shows UQCC1 specifically associates with newly synthesized cytochrome *b* in HEK293 cells. (D) Inhibition of mitochondrial translation in HEK293 cells results in diminished levels of UQCC1, UQCC2, mtDNA-encoded COX1, but does not affect the SDHA subunit of the nuclear encoded complex II.

Having established that UQCC1 and UQCC2 are involved in complex III assembly, we next investigated whether they are involved specifically in cytochrome *b* biogenesis. Mitochondrial translation products from patient and control fibroblasts were subjected to a ^35^S-pulse-chase assay and analyzed by SDS-PAGE. Even at zero hours chase, a striking and specific defect in cytochrome *b* protein levels was observed; other mtDNA-encoded subunits were present in normal amounts or, in the case of COX2 and COX3, an increased amount (Figure 8A). qRT-PCR analysis revealed that *MT-CYB* mRNA levels were unaffected in patient cells (Figure 8B). To determine whether UQCC1 is involved in the stabilization of newly synthesized cytochrome *b*, the UQCC1-TAP purification was carried out with ^35^S metabolically labeled mitochondrial translation products. UQCC1 specifically associated with newly synthesized cytochrome *b* and not with other newly-translated mtDNA-encoded subunits (Figure 8C). Moreover, inhibition of mitochondrial translation with chloramphenicol for 72 h predictably led to the disappearance of mtDNA-encoded COX1, but also UQCC1 and UQCC2 proteins, suggesting that UQCC1 and UQCC2 may be stabilized by cytochrome *b* (Figure 8D). In contrast, SDHA, a nuclear-encoded subunit of complex II, was not affected. We conclude that UQCC1 and UQCC2 are critical factors required for the expression of cytochrome *b* and complex III biogenesis.

## Discussion

Here we report the first case of complex III deficiency due to *UQCC2* mutations. MitoExome MPS identified 3 genes with potentially pathogenic recessive-type variants, of which *UQCC2* was strongly linked to complex III by evolutionary and computational analyses. We demonstrated a *UQCC2* splicing defect resulting in a lack of UQCC2 protein and verified that this gene was causal by restoring complex III protein and activity levels in patient fibroblasts with lentiviral transduction of *UQCC2*. This patient was a singleton from a consanguineous family, and would have been slower to solve using only traditional methods such as homozygosity mapping, which identified 1894 candidate genes in regions of LCSH, at least 86 of which have a putative role in the mitochondria [31]. MitoExome sequencing has already shown promise for the molecular diagnosis of patients with mitochondrial disease [20], [32], [33] and is likely to aid diagnoses in years to come.

Patients with complex III deficiency present with great clinical heterogeneity. Symptoms typically provide little insight into the underlying genetic cause. Our patient shared some of the clinical features of previously reported complex III deficient patients such as tubulopathy and primary lactic acidosis with *BCS1L* patients [9] but only identification of other pathogenic *UQCC2* mutations in unrelated individuals will provide a complete picture of the clinical spectrum of patients with UQCC2 dysfunction.

Complex III deficiencies are commonly accompanied by a reduction in complex I and sometimes complex IV activity [16]–[18]. We also observed reduced presence and activity of complex I in muscle and fibroblasts from P^UQCC2^, which prompted us to provide additional evidence that both UQCC1 and UQCC2 are in fact assembly factors specific to complex III. One type of evidence comes from the analysis of the presence-absence patterns of the two genes among sequenced genomes. The presence and function of *CBP3* and *CBP6* and their orthologs in fungi that encode complex III but have lost complex I (*S. cerevisiae, Schizosaccharomyces pombe*) substantiate their role in complex III biogenesis. Conversely, orthologs of these assembly factors are absent in *B. hominis*, a species that has lost complexes III–V but still encodes complexes I and II. A relative of *B. hominis*, *Phytophthora infestans*, which encodes complex III also encodes an ortholog of Cbp3p/UQCC1 (Figure 3). Coevolution of orthologs of UQCC1 and UQCC2 with complex III and not with complex I indicates that these assembly proteins function primarily in the assembly of complex III.

**Figure 9:**
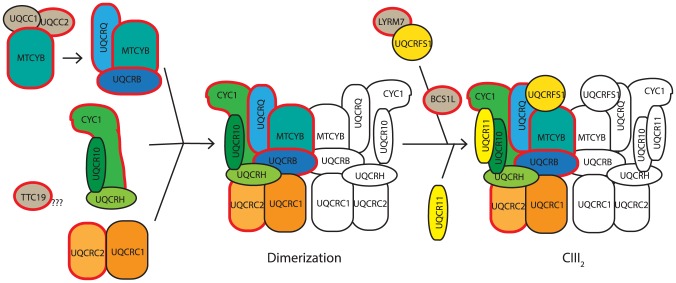
Proposed model of CIII assembly. Complex III assembly begins with the translation activation and/or stabilization of cytochrome *b* (MTCYB) by UQCC1:UQCC2, which then delivers MTCYB to an assembly intermediate containing UQCRQ and UQCRB. This module combines with a module containing CYC1, UQCRH and UQCR10 and a module containing UQCRC2 and UQCRC1. The resulting subcomplex then dimerizes. UQCRFS1 is bound and stabilized by the CIII assembly factor LYRM7, before being incorporated into CIII with the aid of the assembly factor, BCS1L. Finally UQCR11 is added, forming the complete CIII_2_. Assembly factors are indicated in gray. Proteins in which mutations are associated with complex III deficiency are bordered in red. The role of TTC19 is yet to be elucidated, although it is likely to be involved in early complex III assembly. Model adapted and updated from [67].

We elucidated the role of UQCC2, in cooperation with UQCC1, in human cytochrome *b* biogenesis and subsequent complex III assembly and function. No direct link between these proteins and human complex III has previously been described. Previous studies of human UQCC2 suggested that the protein localizes to mitochondrial nucleoids [25] and that it modulates respiratory chain activity in skeletal muscle and pancreatic cells [34]. However, we found no disturbance of mitochondrial nucleoids or mtDNA copy number in patient fibroblasts. Previous studies of human UQCC1 have all focused on variation at this locus being associated with human height [35]–[37]. Our data suggest that UQCC1 and UQCC2 are interacting and interdependent proteins, with the stability of UQCC2 depending on UQCC1, and *vice versa*. We also show that UQCC1 plays an important role in early complex III biogenesis via interaction with newly synthesized cytochrome *b* and recruitment of this mtDNA-encoded subunit into a complex III assembly intermediate (Figure 9).

The direct effect of UQCC1 and UQCC2 dysfunction is an immediate lack of cytochrome *b* leading to disruption of the downstream complex III assembly process. Mutations in *UQCC2* (Figure 5) and UQCC1 depletion (Figure 7) both lead to reduced levels of the complex III subunits UQCRFS1, UQCRC1 and UQCRC2. A subcomplex containing UQCRC1 but not UQCRFS1 accumulates upon *UQCC1* knockdown, consistent with a defect early in CIII assembly before incorporation of the UQCRFS1 subunit (Figure 9). The role of UQCC1:UQCC2 in the initial stages of complex III assembly is further supported by the UQCC1 binding to newly synthesized cytochrome *b* (Figure 8C), although we have not shown that UQCC1 and UQCC2 bind to cytochrome *b* together.

Interestingly, in *S. cerevisiae*, Cbp3p and Cbp6p have been shown to provide a feedback loop modulating cytochrome *b* expression in response to complex III assembly [30]. Cbp6p and Cbp3p, bind to *MT-CYB* mRNA to activate its translation and then deliver newly-synthesized cytochrome *b* to a complex III assembly intermediate. When early complex III assembly is disrupted, cytochrome *b* cannot be deposited by the Cbp3p:Cbp6p complex and so these factors remain bound to the cytochrome *b* protein. While bound in an assembly intermediate, the Cbp3p:Cbp6p complex is unable to activate further cytochrome *b* translation, thus modulating cytochrome *b* synthesis in response to complex III assembly. Such coordination between mtDNA translation and nuclear gene expression prevents the build-up of mtDNA-encoded proteins in the absence of functional complexes. It will be interesting to investigate whether UQCC1 and UQCC2 provide a similar feedback loop between cytochrome *b* translation and complex III assembly in mammalian mitochondria.

The role of UQCC1 and UQCC2 in cytochrome *b* expression like their *S. cerevisiae* orthologs Cbp3p and Cbp6p, is supported by the fact that no cytochrome *b* synthesis is detected in P^UQCC2^ via a mitochondrial translation assay (Figure 8A). Furthermore, mitochondrial translation is a prerequisite for the stability and function of both proteins (Figure 8D). The previously reported co-localization of UQCC2 with mitochondrial nucleoids would be consistent with a role in mitochondrial translation, as factors required for mitochondrial protein synthesis, such as ATAD3 and PHB, are often found to associate with mitochondrial nucleoids [38]. Nevertheless, whether UQCC1 and UQCC2 are directly required for translation activation of *MT-CYB* remains to be established, specifically because the 5′ UTRs of mitochondrial mRNAs to which translational activators in *S. cerevisiae* bind are absent from human mitochondrial mRNAs. Furthermore, in the fungus *S. pombe* the function of the Cbp6p ortholog appears to be only post-translational [39]. One can speculate that the lack of sequence conservation of the N-terminus of the Cbp3p orthologs, even among fungi, could explain the lack of conservation of translation activation. Nevertheless, there is currently no information about which region of *S. cerevisiae* Cbp3p is required for translation activation and the N-terminus of Cbp3p appears to be relatively dispensable for its function, even in *S. cerevisiae* itself [28]. To date, only one putative human mitochondrial translational activator, TACO1, which is required for the translational activation of the COX1 subunit of complex IV, has been described [40].

In summary, here we have used MitoExome MPS in combination with computational and experimental analyses to identify the first case of complex III deficiency due to *UQCC2* mutation. We demonstrate that UQCC2 and its binding partner UQCC1 are required for early complex III assembly by mediating the synthesis, stability and/or assembly of the mtDNA-encoded complex III subunit, cytochrome *b*.

## Materials and Methods

### Ethics statement

Investigations were performed with ethics approval by the Human Research Ethics Committee of the Royal Children's Hospital, Melbourne.

### Patient clinical summary

The proband was the first child of first cousin Lebanese parents and was patient P12 in [20]. The pregnancy was complicated by intrauterine growth retardation, and he was born at 36 weeks gestation by emergency caesarean section because of placental compromise. Birth weight was 1280 gm, length 41 cm and head circumference 29 cm. He had good Apgar scores (9 and 9 at one and five minutes respectively), but by 12 hours of age he became lethargic and had loose stools. He was found to have severe metabolic acidosis (pH 7.16, lactate 9.6 mmol/L; normal range 0.7–2.0), and CSF lactate at around that time was 3.8 mmol/L (normal <2.0). His blood electrolytes suggested he had a proximal renal tubular acidosis. His condition improved with rehydration and bicarbonate supplementation, but blood lactate remained high (5–14 mmol/L) even when well. In addition, he was mildly dysmorphic with synophrys, epicanthic folds, upward slanting palpebral fissures, a depressed nasal bridge and flattened nose, and had a unilateral undescended testis. He also had unilateral postaxial polydactyly, the skin tag being removed in the newborn period. His father had a similar facial appearance and had a history of a cleft palate. Seizures were effectively treated with phenobarbitone for 6 months, after which it was ceased. A CT and MRI scan of the brain revealed no abnormality. A vitamin cocktail including riboflavin, thiamine, vitamin C, biotin and coenzyme Q did not appear to have an effect on his blood lactate levels or clinical condition.

He developed acute gastroenteritis at 5 months of age, at which time severe metabolic acidosis again developed, which again resolved with rehydration. Afebrile seizures recurred at two years of age, and were treated with sodium valproate and later with lamotrigine. EEG was normal at this time.

Developmental milestones were delayed: he sat unaided at six months, crawled at 10 months, could cruise around furniture at 13 months, walked unaided at 15 months, but still had no formal speech by 2 years 3 months. By that age he could walk unaided, but frequently fell. Stamina was normal. Formal neuropsychological review at two years nine months revealed severe delay in fine motor and visuo-spatial performance, self-care skills and social play, with gross motor skills being only mildly impaired. There were no concerns with vision, but he had a mild sensorineural hearing impairment. Despite speech therapy, at six years of age he still had only several words in Arabic but no meaningful speech.

He went on to develop a number of autistic features, including impulsivity, limited eye contact, extreme hyperactivity, aggressive behaviour, night time roaming, and continued to have no real expressive language, although he was felt to have reasonable receptive language. He required two minders at school because of concerns of his lack of regard for his physical safety. Ritalin caused him to become even more agitated, whereas clonidine appeared to be of some benefit. He was lost to follow up at nine years of age.

### General methods

Unless otherwise described below, cell culture, DNA isolation, RNA isolation, cDNA synthesis and sequencing of PCR products were performed as described previously [41]. To sequence unique splice variants, RT-PCR products were first cloned into a pTOPO vector using the TOPO TA Cloning Kit (Invitrogen) as per manufacturer's protocol. For mitochondrial nucleoid staining, patient fibroblasts and control cell lines were grown on coverslips and stained with 3 µl/ml PicoGreen [42] (Invitrogen) for 1 hour and 10 nM MitoTracker Red CMXRos (Invitrogen) for 30 min at 37°C, 5% CO_2_. Coverslips were washed with PBS, then mounted on slides for live cell imaging using a Zeiss AxioImager.M1 epifluorescence microscope.

### MitoExome sequencing

Genes encoding the entire predicted mitochondrial proteome (1381 nuclear genes and the mtDNA) were captured and sequenced on an Illumina Genome Analyzer II as described previously [20]. Variant prioritization used previously established criteria for likely pathogenicity [20], [41].

### Biochemical analysis

Spectrophotometric enzyme assays assessing mitochondrial OXPHOS activity were performed as described previously for patient samples (muscle post-nuclear supernatants and fibroblast mitochondria) [43] and for functional studies in mitochondria from HEK293 cells, see [44] and references therein.

### Iterative orthology prediction

An orthology identification pipeline that uses sequences, sequence-based profiles as well as profile-derived Hidden Markov Models [26] was applied to identify human orthologs of fungal Cpb3p and Cbp6p proteins. As a negative control for the orthology prediction method, sequence-based profiles of the orthologs were also used to search for orthologs in the genome nucleotide sequence of *B. hominis* [45], a species with mitochondria-like organelles but without complex III. No orthologs were found in the *B. hominis* genome despite using sensitive PSI-tblastn [46] to circumvent possible gene annotation errors.

### mRNA co-expression analysis

To calculate the Pearson correlation of mRNA expression in murine tissues and cell types, 182 microarray sample measurements with Affymetrix Mouse Genome 430 2.0 Array [47] were used. The data (GNF Mouse GeneAtlas V3) were downloaded from Gene Expression Omnibus, record GSE10246 [47]. The data were transformed as described previously [48].

### qRT-PCR

Quantitative expression analysis of *UQCC2* was performed as previously described [49], using the *MNF1* Hs00942667_m1 gene expression assay (Life Technologies) that detects the exon 2/3 junction of *UQCC2 (MNF1)* with the *HPRT1* Endogenous Control Gene Expression Assay (Life Technologies) for normalization, and the *Cytb* (*MT-CYB*) Hs02596867_s1 gene expression assay (Life Technologies) with the previously described ND1 assay [50] for normalization. Because the *Cytb* and *ND1* assays cannot distinguish cDNA from mtDNA, an additional DNAse-treatment was performed prior to qRT-PCR using the Turbo DNA-free kit (Ambion) as per manufacturer's protocol.

Quantitative analysis of mtDNA copy number was performed with a probe targeting *ND1* to represent mtDNA and a probe targeting *CFTR* as the nuclear reference, as described previously [51].

### SNP analysis

Molecular karyotyping of patient DNA was performed with the Illumina HumanCytoSNP-12 array (version 2.1) as previously described [52]. Automated LCSH detection was performed with the CNVPartition v3.1.6 algorithm in KaryoStudio software. SNP genotypes were generated in GenomeStudio software (Illumina) with data from a set of 102 intra-run samples.

### Sequenom genotyping

A Sequenom assay using multi-plexed MALDI-TOF mass spectrometry was designed to genotype 86 Lebanese controls for the c.214-3C>G mutation. The forward, reverse and extension primers were as follows: 5′ACGTTGGATGCTTCACTTCCTTTCTGCCCC3′, 5′ACGTTGGATGTGTACTCTTCCAACGACAGG3′, 5′CACTTCCTTTCTGCCCCGGTGAC3′. Genotypes were called using the MassARRAY System Typer version 4.0 (Sequenom).

### Viral particle production and transduction

Full length *UQCC2* was amplified from cDNA using high-fidelity Phusion Taq (Finnzymes) with a forward primer incorporating a 5′ BamHI recognition site (5′CGGGATCCACCATGGCGGCCAGCCGGTACCGGCGTT3′) and a reverse primer incorporating a 3′ XbaI recognition site (5′GCTCTAGATTATCAGGCCTTATGATCCTCCTCAGGAC3′). The resulting RT-PCR product was cloned into the 4-hydroxytamoxifen-inducible lentiviral vector, pF_5x_UAS_MCS_SV40_puroGEV16-W [53]. UQCC2 viral particles were generated and patient fibroblasts were transduced as described previously [41]. Three independent transductions were performed and cells were harvested 12–18 days after selection with 1 mg/ml puromycin.

### SDS-PAGE and BN-PAGE

One-dimensional 5–15% BN gradient and two-dimensional (2D) SDS gradient PAGE were done as described previously [54], [55]. Whole cell or tissue samples were used for SDS-PAGE and isolated mitochondria were used for BN-PAGE. SDS-PAGE with 10% NuPage gels (Invitrogen) and immunoblotting was performed as described previously [41]. Proteins were detected with the following antibodies: α-MNF1 (ATLAS antibodies or as previously described [25] for detection of UQCC2), Total OXPHOS Human WB Antibody Cocktail containing α-ATP5A1, α-UQCRC2, α-SDHB, α-COX2 and α-NDUFB8 (MitoSciences), α-NDUFA9 (as previously described [55]), α-UQCC (Atlas Antibodies), α-CBP (GenScript), α-ND1 (kindly provided by A Lombes [56]), α-cyclophilin B (Affinity Bioreagents), α-OXA1L (Central Animal Facility Nijmegen), α-SDHA, α-SDHB, α-COX1, α-UQCRC1, α-UQCRC2, α-UQCRFS1 and α-ATP5α (all MitoSciences), α-TOM20 (BD transduction laboratories), α-CK-B 21E10 (kindly provided by the Department of Cell Biology Nijmegen [57]), α-MRPL12 (Abcam), α-MRPS22 (Proteintech) and α-VDAC1 (Calbiochem). Secondary antibodies were goat α-mouse or swine α-rabbit IgG horseradish peroxidase (HRP, DakoCytomation), goat α-mouse or α-rabbit IgG HRP antibodies (Invitrogen). Quantification of western blots was performed by densitometry using ImageJ software or the Chemidoc XRS+ system (Biorad) software.

### Mitochondrial translation assay

zMitochondrial translation assays were performed as described previously [58], [59]. Briefly, fibroblasts were cultured with cycloheximide to inhibit cytoplasmic translation and mtDNA-encoded proteins were labeled with a 2-hour pulse of ^35^S-methionine/^35^S-cysteine (EXPRE^35^S^35^S Protein Labeling Mix; Perkin Elmer Life Sciences) prior to washing and a chase with cold methionine for 0 to 24 hours. Mitochondria were then isolated and translation products were analyzed by SDS-PAGE and autoradiography. HEK293 cells were cultured and labeled in the same way, except that labeling was done for 1 hour, emetine was used instead of cycloheximide and Tran^35^S-Label (MP Biomedicals) was used for labeling.

### Statistics

Two-way repeated-measures analysis of variance (ANOVA) was used for comparisons of groups followed by post hoc analysis with a Bonferroni correction to account for multiple comparisons.

### Cloning and generation of UQCC1 and UQCC2 expression plasmids

The *UQCC2* open reading frame was PCR amplified without the stop codon from HEK293 cDNA adding Attb recombination sites (underlined) using the following primers: forward 5′-AA AAAGCAGGCTTCGCCACC ATGGCGGCCAGCCGGTACCGGCG-3′ and reverse 5′-GAAAGCTGGGTG GGCCTTATGATCCTCCTCAGG-3′. After the first PCR, the specific product was used in a second PCR using this primer set: forward 5′-GGGGACAAGTTTGTACAAAAAAGCAGGCT-3′ and reverse 5′-GGGGACCACTTTGTACAAGAAAGCTGGGT-3′ to complete the recombination sites and allow the cloning in the pDONR201 vector using BP clonase enzyme mix (Invitrogen). The pDONR201 vector containing the *UQCC1* open reading frame without stop codon was obtained from the Harvard Medical School (clone ID: HsCD00081684) [60]. Next, mammalian expression vectors under the control of a tetracycline-inducible promoter adding a tandem affinity purification (TAP) tag at the C-terminus were generated by recombining the pDONR201 vectors with the appropriate expression vector with the aid of LR clonase enzyme mix. All vectors were checked with sequence analysis before further use.

### Cell culture

T-REx Flp-In Human Embryonic Kidney 293 cells (HEK293; Invitrogen) were grown and maintained in Dulbecco's Modified Eagles Medium (DMEM; Biowhitaker) supplemented with 10% FCS, 1% [v/v] penicillin/streptomycin, zeocin (300 µg/ml, Invitrogen) and 5 µg/ml blasticidin (Calbiochem). To generate stable cell lines expressing TAP fusion proteins, cells were transfected with the corresponding construct using Superfect transfection reagent (Qiagen) and selected for stable transfectants by replacing the zeocine in the culture medium with hygromycin (200 µg/ml, Calbiochem). Gene expression was induced by adding 1 µg/ml doxycycline (Sigma) to the culture medium for a minimum of 24 h. Mitochondrial translation was inhibited with 40 µg/ml chloramphenicol (CAP) for a minimum of 72 h.

### siRNA design and transfections

siRNAs were designed using the online available software from the Whitehead Institute for Biomedical Research [61] and synthesized by Biolegio (Nijmegen). The following siRNAs were used: *UQCC2* antisense 5′-AGUAGUUUGAAUGGAGUCG dTdT-3′; *UQCC1* antisense 5′-UAUGAUACGACACAUGUAC dTdT-3′ as well as control *cyclophilin B* targeting siRNA (Thermo Scientific). For transfection HEK293 cells were plated in antibiotic-free culture medium and transfected the next day with 10 nM siRNAs using Dharmafect 1 transfection reagent (Dharmacon). At day 3, cells were split 1∶4 and transfected again the next day. Cells were harvested 96 hours after the first transfection and analyzed with SDS and/or BN-PAGE.

### Cellular fractionation and proteinase K protection assay

The cellular fractionation of HEK293 cells was done as previously described [62]. For determining the submitochondrial localization a proteinase K protection assay was performed as previously described [63].

### Isolation of mitoplasts and determination of protein concentrations

Mitoplasts were pelleted by centrifugation as described before [64]. The supernatants containing the solubilized proteins were used for further analysis. Protein concentrations of the samples were determined with the microBCA protein kit (Thermo Scientific).

### Single step affinity purifications

HEK293 cells were induced with doxycycline for 24 h to express UQCC1-TAP or UQCC2-TAP fusion proteins before being harvested and processed for a single step affinity purification using the Interplay Mammalian TAP kit (Agilent Technologies) as per manufacturer's protocol.

## Supporting Information

Figure S1 Analysis of a 300K Illumina SNP array showing long contiguous stretches of homozygosity (LCSH) on chromosome 6. The position of the *UQCC2* gene in a 30.7 kb region of LCSH is indicated by a dashed line. These data support the mutation being homozygous due to identity by descent i.e., inheritance of both alleles from a common ancestor.
(PDF)

Figure S2 The c.214-3C>G *UQCC2* site has moderate conservation in vertebrates. Alignment of vertebrate *UQCC2* gDNA sequence around the site of the c.214-3C>G *UQCC2* mutation (bordered in red). The consensus AG acceptor site is bordered in black.
(PDF)

Figure S3 The c.214-3C>G mutation results in no detectable UQCC2 protein. Western blot shows a lack of UQCC2 protein in P^UQCC2^, and no truncated or elongated protein that might be encoded by the alternative splice species (predicted sizes of 11.3 kDa and 18.7 kDa respectively). Asterisk indicates a non-specific band.
(PDF)

Figure S4 Mitochondrial nucleoids and mtDNA are undisturbed in P^UQCC2^. (A) Fibroblasts from Controls (C^1^–C^2^), P^UQCC2^ and P^POLG^, were stained with PicoGreen to visualize mitochondrial nucleoids and cellular nuclei (top panels), and MitoTracker Red (middle panels) to visualize mitochondrial networks. Merged images (bottom panels) indicate alignment of nucleoids with mitochondrial networks. Nucleoids from P^POLG^ are poorly stained and few in number or absent from cells, whereas nucleoids from P^UQCC2^ are similar in number and distribution to control cells. (B) MtDNA copy number was determined using qPCR targeting *ND1* on mtDNA and *CFTR* as the nuclear reference. Three independent assays were performed, each in triplicate. Bars represent average ND1:CFTR ratios relative to 4 control fibroblast lines ±1 s.e.m.
(PDF)

Figure S5 Additional evidence of complex III restoration in P^UQCC2^ with *UQCC2* transduction. (A) Spectrophotometric enzyme analysis shows a clear increase in CIII activity in P^UQCC2^ but not in wild-type control cells, following transduction with *Lv-UQCC2*. (B) BN-PAGE of digitonin-lysed mitochondria shows a lack of complex III dimer and minimal complex III bound in supercomplexes, and restoration of normal ratios following *UQCC2* transduction. (C) SDS-PAGE immunoblotting shows a lack of UQCC1 protein in P^UQCC2^ and restoration following transduction with *UQCC2*.
(PDF)

Figure S6 Quantification of protein expression in patient fibroblasts. Protein expression was visualized with immunoblots and quantified by densitometry. Y-axis shows relative intensities of proteins of interest normalized to loading controls, expressed as a percentage of the control. (A) BN-PAGE immunoblot analysis of holocomplexes from Figure 5A. (B) SDS-PAGE immunoblot analysis of complex III subunits, UQCC1 and UQCC2 from Figure 5B.
(PDF)

Figure S7 Quantification of protein/complex expression in UQCC1 and UQCC2 knockdown experiments. Y-axis shows relative intensities of the immunoreactive bands compared to mock transfection (100 = no change). (A) SDS-PAGE immunoblot analysis of UQCC2 knockdown from Figure 5C. (B) SDS-PAGE immunoblot analysis of UQCC1 knockdown from Figure 7A. (C) Blue Native PAGE immunoblot analysis of UQCC1 knockdown from Figure 7B.
(PDF)

Figure S8 Additional biochemical analysis of UQCC1 depleted cells. (A) SDS-PAGE and Western blot analysis of mitochondrial extracts from HEK293 cells transfected with siRNAs targeting *UQCC1*, with a separate set of antibodies than were used for Figure 7A. Cyclophilin B and mock transfected cells were used as control. Loss of UQCC1 results in depletion of UQCC2 and reduced levels of UQCRC2 protein levels. Subunit levels of other OXPHOS-complexes: NDUFB8 (complex I), SDHB (complex II), COX2 (complex IV) and ATP5α (complex V) are not affected by UQCC1 knock-down. Antibodies used are indicated at the left. (B) Quantification of the immunoreactive bands is shown at the right.
(PDF)

Text S1 Detailed analysis of the splice variants in P^UQCC2^.
(DOCX)
