# Supplementary figures and images for: Mutations in the UQCC1-Interacting Protein, UQCC2, Cause Human Complex III Deficiency Associated with Perturbed Cytochrome b Protein Expression
Source: PLoS Genet. 2013 Dec 26;9(12):e1004034. doi: 10.1371/journal.pgen.1004034 (PMC3873243; doi:10.1371/journal.pgen.1004034)

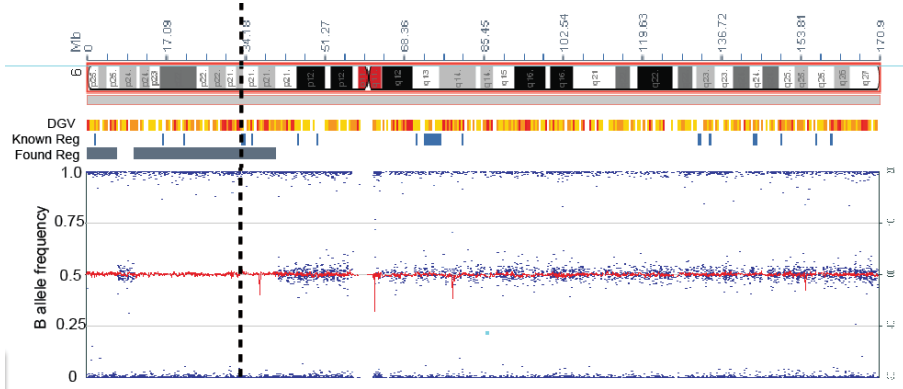

Supplement: Figure S1 — Analysis of a 300K Illumina SNP array showing long contiguous stretches of homozygosity (LCSH) on chromosome 6. The position of the UQCC2 gene in a 30.7 kb region of LCSH is indicated by a dashed line. These data support the mutation being homozygous due to identity by descent i.e., inheritance of both alleles from a common ancestor. (PDF) [file pgen.1004034.s001.pdf]

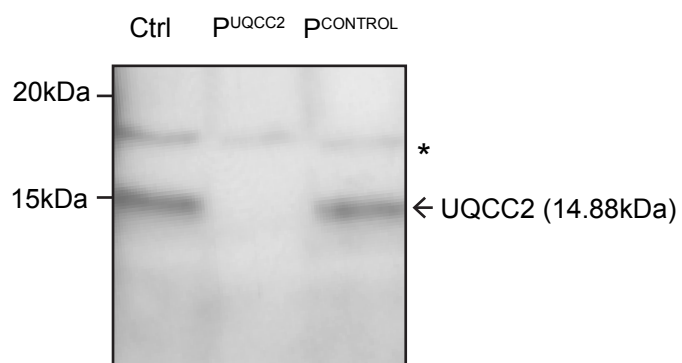

Supplement: Figure S3 — The c.214-3C>G mutation results in no detectable UQCC2 protein. Western blot shows a lack of UQCC2 protein in PUQCC2, and no truncated or elongated protein that might be encoded by the alternative splice species (predicted sizes of 11.3 kDa and 18.7 kDa respectively). Asterisk indicates a non-specific band. (PDF) [file pgen.1004034.s003.pdf]

**A**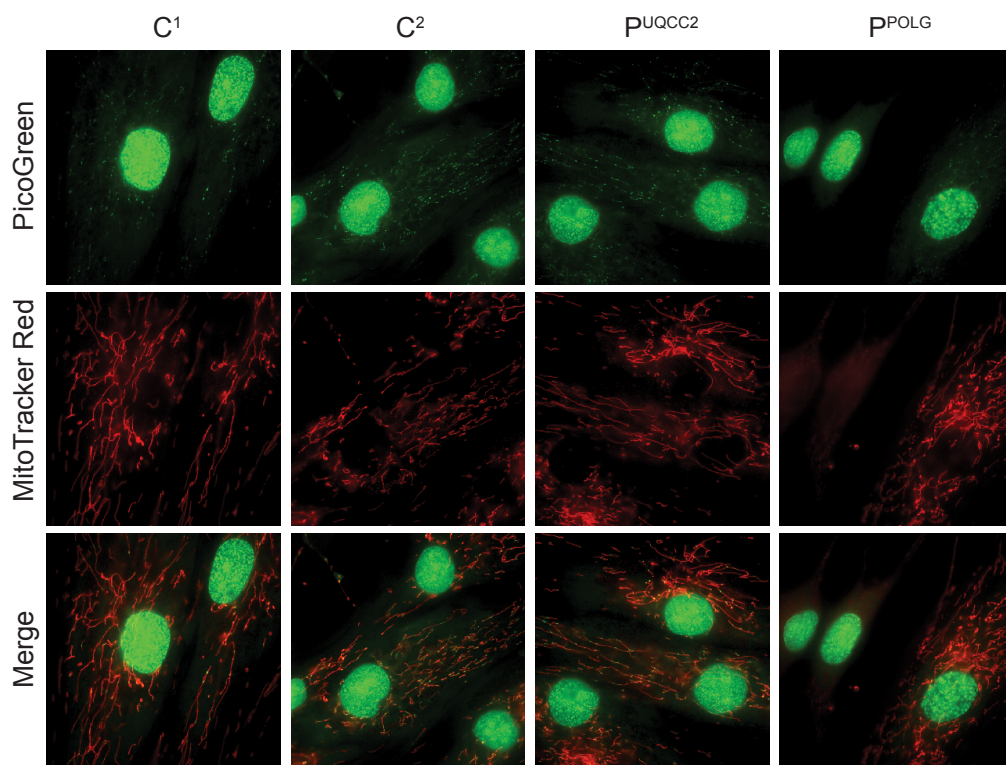**B**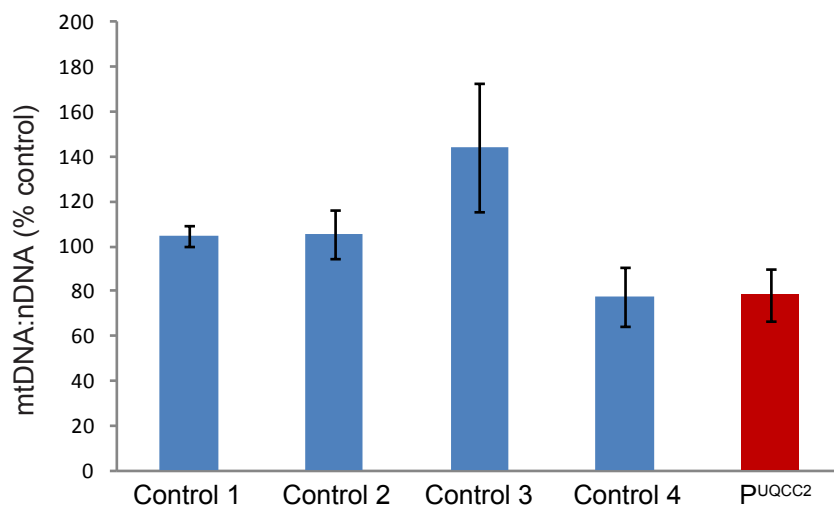

Supplement: Figure S4 — Mitochondrial nucleoids and mtDNA are undisturbed in PUQCC2. (A) Fibroblasts from Controls (C1–C2), PUQCC2 and PPOLG, were stained with PicoGreen to visualize mitochondrial nucleoids and cellular nuclei (top panels), and MitoTracker Red (middle panels) to visualize mitochondrial networks. Merged images (bottom panels) indicate alignment of nucleoids with mitochondrial networks. Nucleoids from PPOLG are poorly stained and few in number or absent from cells, whereas nucleoids from PUQCC2 are similar in number and distribution to control cells. (B) MtDNA copy number was determined using qPCR targeting ND1 on mtDNA and CFTR as the nuclear reference. Three independent assays were performed, each in triplicate. Bars represent average ND1:CFTR ratios relative to 4 control fibroblast lines ±1 s.e.m. (PDF) [file pgen.1004034.s004.pdf]

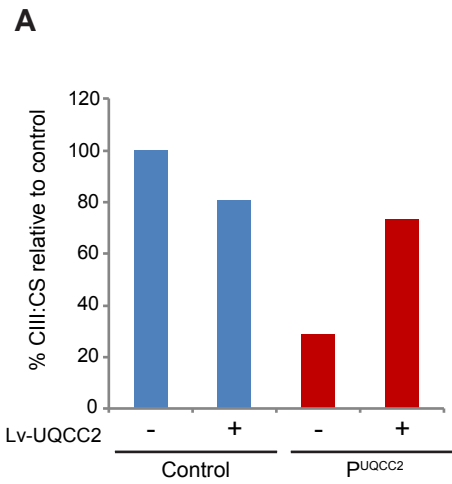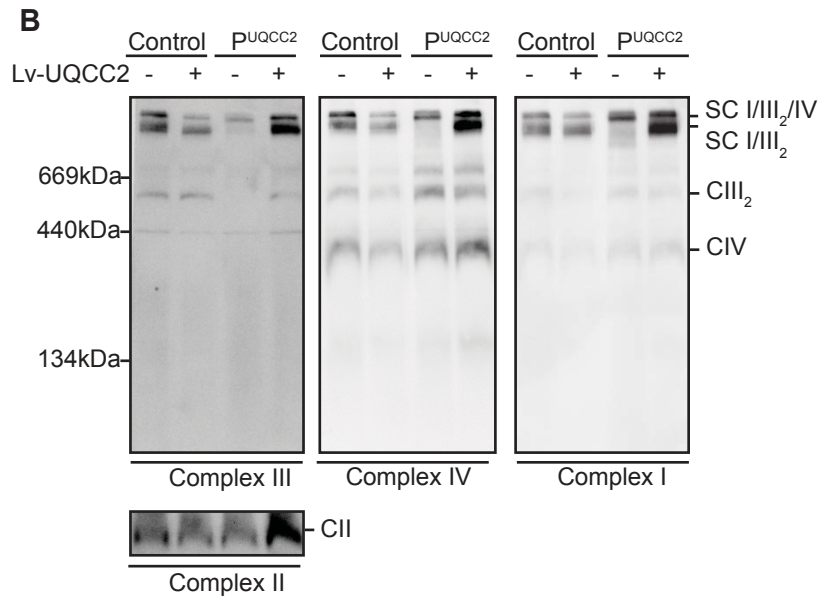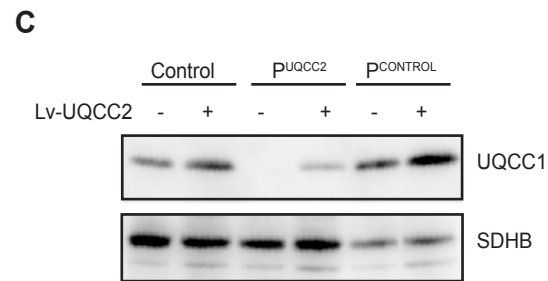

Supplement: Figure S5 — Additional evidence of complex III restoration in PUQCC2 with UQCC2 transduction. (A) Spectrophotometric enzyme analysis shows a clear increase in CIII activity in PUQCC2 but not in wild-type control cells, following transduction with Lv-UQCC2. (B) BN-PAGE of digitonin-lysed mitochondria shows a lack of complex III dimer and minimal complex III bound in supercomplexes, and restoration of normal ratios following UQCC2 transduction. (C) SDS-PAGE immunoblotting shows a lack of UQCC1 protein in PUQCC2 and restoration following transduction with UQCC2. (PDF) [file pgen.1004034.s005.pdf]

**A**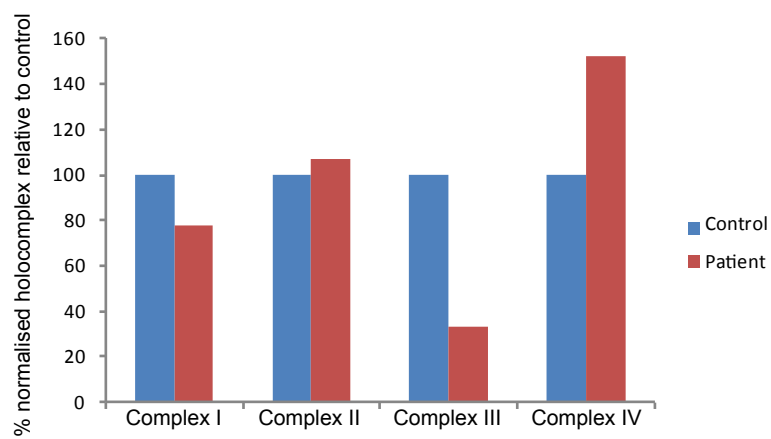**B**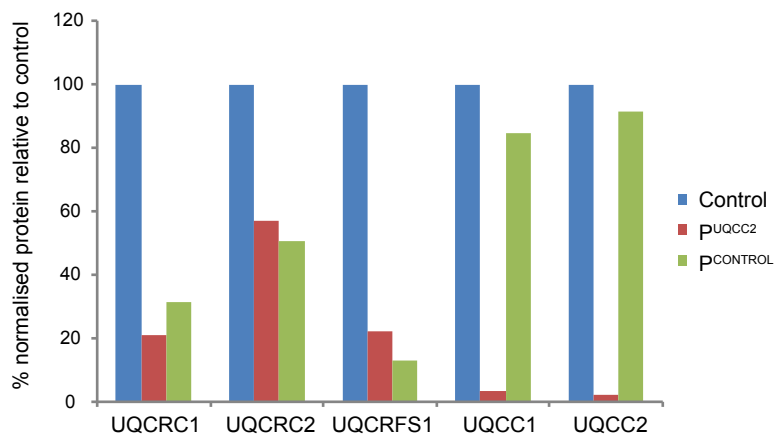

Supplement: Figure S6 — Quantification of protein expression in patient fibroblasts. Protein expression was visualized with immunoblots and quantified by densitometry. Y-axis shows relative intensities of proteins of interest normalized to loading controls, expressed as a percentage of the control. (A) BN-PAGE immunoblot analysis of holocomplexes from Figure 5A. (B) SDS-PAGE immunoblot analysis of complex III subunits, UQCC1 and UQCC2 from Figure 5B. (PDF) [file pgen.1004034.s006.pdf]

**A**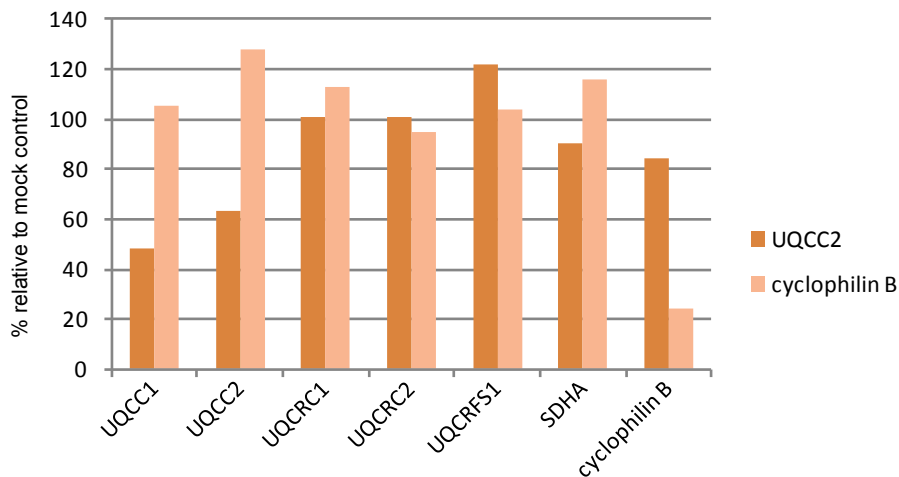**B**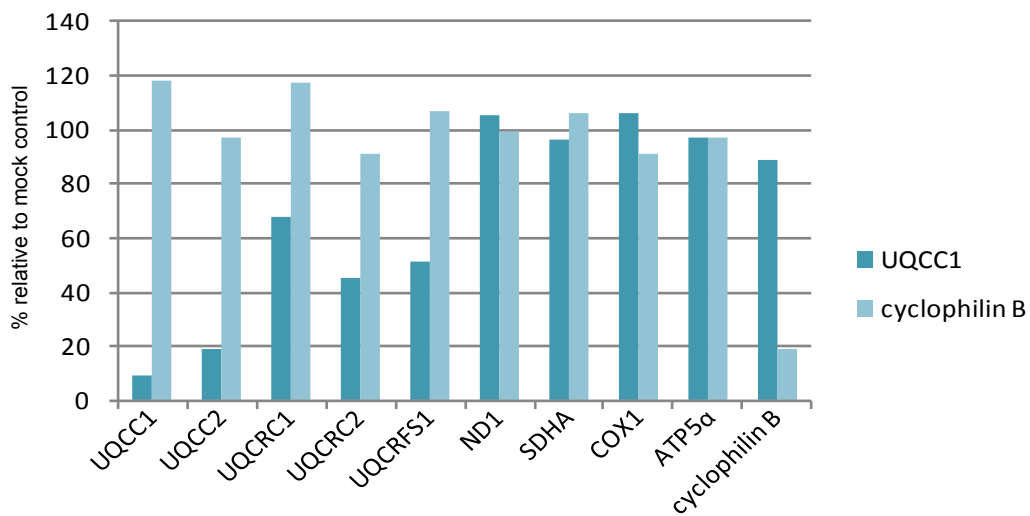**C**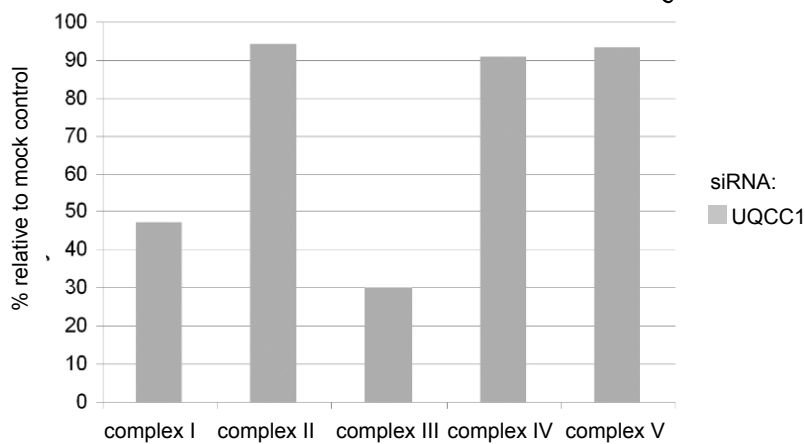

Supplement: Figure S7 — Quantification of protein/complex expression in UQCC1 and UQCC2 knockdown experiments. Y-axis shows relative intensities of the immunoreactive bands compared to mock transfection (100 = no change). (A) SDS-PAGE immunoblot analysis of UQCC2 knockdown from Figure 5C. (B) SDS-PAGE immunoblot analysis of UQCC1 knockdown from Figure 7A. (C) Blue Native PAGE immunoblot analysis of UQCC1 knockdown from Figure 7B. (PDF) [file pgen.1004034.s007.pdf]

**A**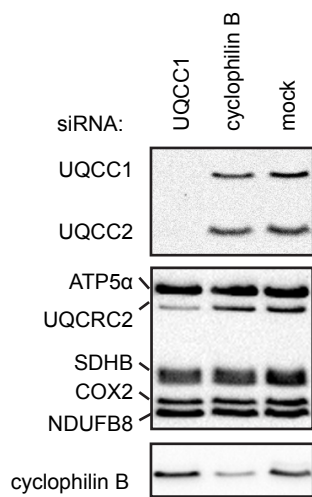**B**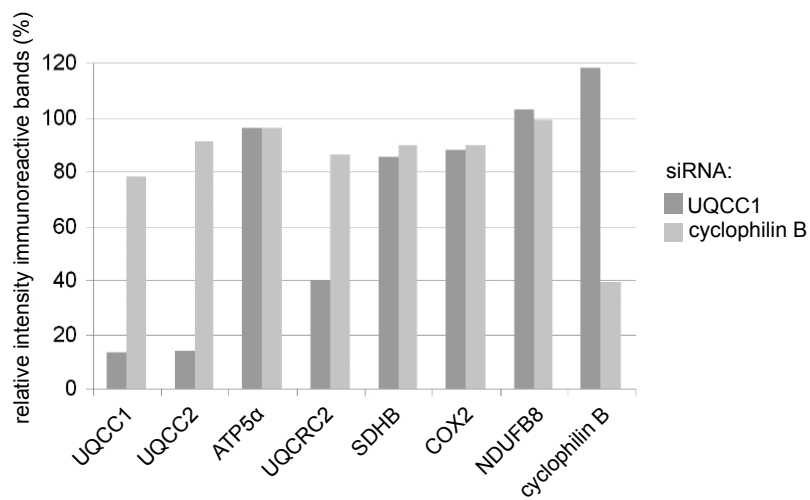

Supplement: Figure S8 — Additional biochemical analysis of UQCC1 depleted cells. (A) SDS-PAGE and Western blot analysis of mitochondrial extracts from HEK293 cells transfected with siRNAs targeting UQCC1, with a separate set of antibodies than were used for Figure 7A. Cyclophilin B and mock transfected cells were used as control. Loss of UQCC1 results in depletion of UQCC2 and reduced levels of UQCRC2 protein levels. Subunit levels of other OXPHOS-complexes: NDUFB8 (complex I), SDHB (complex II), COX2 (complex IV) and ATP5α (complex V) are not affected by UQCC1 knock-down. Antibodies used are indicated at the left. (B) Quantification of the immunoreactive bands is shown at the right. (PDF) [file pgen.1004034.s008.pdf]
