# Supplementary material for: Mutations in the UQCC1-Interacting Protein, UQCC2, Cause Human Complex III Deficiency Associated with Perturbed Cytochrome b Protein Expression
Source: PLoS Genet. 2013 Dec 26;9(12):e1004034. doi: 10.1371/journal.pgen.1004034 (PMC3873243; doi:10.1371/journal.pgen.1004034)
Supplement: Figure S2 — The c.214-3C>G UQCC2 site has moderate conservation in vertebrates. Alignment of vertebrate UQCC2 gDNA sequence around the site of the c.214-3C>G UQCC2 mutation (bordered in red). The consensus AG acceptor site is bordered in black. (PDF) [file pgen.1004034.s002.pdf]

Homo sapiens	g	t	g	a	c	c	a	g	T	A	C	C	C	T	C	G	C
Pan troglodytes	g	t	g	a	c	c	a	g	T	A	C	C	C	T	C	G	C
Pongo pygmaeus abelii	g	t	g	a	c	c	a	g	T	A	C	C	C	T	C	G	C
Macaca mulatta	g	t	g	a	c	c	a	g	T	A	C	C	C	T	C	G	C
Papio hamadryas	g	t	g	a	c	c	a	g	T	A	C	C	C	T	C	G	C
Callithrix jacchus	g	t	g	a	c	c	a	g	T	A	C	C	C	T	C	G	C
Tarsius syrichta	g	t	g	a	c	c	a	g	T	A	C	C	C	T	C	G	C
Microcebus murinus	g	t	g	a	c	c	a	g	T	A	C	C	C	T	C	G	C
Otolemur garnettii	a	t	g	a	c	t	a	g	T	A	T	C	C	T	C	G	G
Tupaia belangeri	a	c	g	a	c	c	a	g	T	A	C	C	C	T	C	G	C
Cavia porcellus	=	=	=	=	=	c	a	g	T	A	C	C	C	C	C	G	G
Dipodomys ordii	=	=	=	=	=	c	a	g	T	A	T	C	C	T	C	G	C
Mus musculus	=	=	=	=	=	c	a	g	T	A	C	C	C	T	C	G	C
Rattus norvegicus	=	=	=	=	=	=	a	g	T	A	C	C	C	T	C	G	C
Spermophilus tridecemlineatus	=	=	=	=	=	c	a	g	T	A	T	C	C	C	C	G	C
Oryctolagus cuniculus	=	=	=	=	=	c	a	g	T	A	C	C	C	T	C	G	G
Ochotona princeps	=	=	=	=	=	c	a	g	T	A	C	C	C	A	C	G	C
Vicugna pacos	g	t	g	a	c	c	a	g	T	A	C	C	C	T	C	G	C
Tursiops truncatus	g	t	g	a	c	c	a	g	T	A	C	C	C	T	C	G	C
Equus caballus	g	t	g	t	c	c	a	g	T	A	C	C	C	T	C	G	C
Bos taurus	g	t	g	a	c	c	a	g	T	A	C	C	C	T	C	G	C
Felis catus	g	t	g	a	c	c	a	g	T	A	C	C	C	T	C	G	C
Canis lupus familiaris	g	t	g	a	c	c	a	g	T	A	C	C	C	T	C	G	C
Erinaceus europaeus	a	t	g	a	c	t	a	g	T	A	C	C	C	T	C	G	C
Sorex araneus	=	=	=	a	c	t	a	g	T	A	C	C	C	T	C	G	T
Loxodonta africana	g	t	g	a	c	c	a	g	T	A	C	C	C	A	C	G	C
Procavia capensis	t	t	g	g	c	c	a	g	T	A	C	C	C	A	C	G	C
Echinops telfairi	g	t	a	a	c	c	a	g	T	A	C	C	C	T	C	G	C
Dasypus novemcinctus	g	t	g	a	c	c	a	g	T	A	C	C	C	T	C	G	C
Choloepus hoffmanni	g	t	g	a	c	c	a	g	T	A	C	C	C	T	C	G	C
Macropus eugenii	t	t	t	g	t	a	a	g	T	A	T	C	C	A	C	G	C
Monodelphis domestica	t	g	g	g	a	t	a	g	T	A	T	C	C	T	C	G	C
Ornithorhynchus anatinus	c	g	g	c	c	a	a	g	T	A	C	C	C	T	C	G	C
Gallus gallus	-	-	-	-	-	-	a	g	T	A	T	C	C	A	C	G	C
Taeniopygia guttata	=	=	=	=	=	=	a	g	T	A	C	C	C	A	C	G	C
Anolis carolinensis	=	=	=	=	=	=	a	g	T	A	T	C	C	A	C	G	T
