# Supplementary material for: Mutations in the UQCC1-Interacting Protein, UQCC2, Cause Human Complex III Deficiency Associated with Perturbed Cytochrome b Protein Expression
Source: PLoS Genet. 2013 Dec 26;9(12):e1004034. doi: 10.1371/journal.pgen.1004034 (PMC3873243; doi:10.1371/journal.pgen.1004034)
Supplement: Text S1 — Detailed analysis of the splice variants in PUQCC2. (DOCX) [file pgen.1004034.s009.docx]

**Supplementary Text S1: Detailed analysis of the splice variants in P^UQCC2^.**

While only one prominent RT-PCR product was amplified from control cDNA, two prominent bands were detected in patient fibroblasts (Figure 2A). Sequencing revealed that the band with higher molecular weight than the control band corresponded to the use of a cryptic acceptor site, which introduced 108 bases of intronic sequence into the transcript (r.[214-108_214-1ins; 214-3c>g]). This transcript is stable and is predicted to encode a protein with 36 additional amino acids (p.K71_Y72ins36). Sequencing of the band with comparable molecular weight to the control band, revealed that it too used an alternative acceptor site which resulted in a transcript lacking the first 14 bases of exon 3 (r.214_227del). This splice variant is predicted to encode a frame-shifted protein (p.Y72Rfs*25) but the transcript appears stable as it is detected in the absence of cycloheximide, which inhibits nonsense mediated decay. According to *in silico* analysis (Human Splicing Finder v2.4.1 http://www.umd.be/HSF/), the wild-type acceptor site has a predicted score of 78.02 and the mutation reduces this score to 67.73. The upstream and downstream cryptic acceptor sites have scores of 83.74 and 88.95 respectively, consistent with their preferential use in patient fibroblasts.
